# One Notable Complication of Nasopharyngeal Airway: A Case Report

**DOI:** 10.5811/cpcem.2020.8.48811

**Published:** 2020-10-20

**Authors:** Blake Briggs, Chase Countryman, Henderson D. McGinnis

**Affiliations:** * University of South Alabama, Department of Emergency Medicine, Mobile, Alabama; † Wake Forest School of Medicine, Department of Emergency Medicine, Winston-Salem, North Carolina

**Keywords:** Nasopharyngeal airway, nasal foreign body, EMS, emergency department communication, retained foreign bodies

## Abstract

**Introduction:**

The nasopharyngeal airway (NPA) is used by emergency providers and first responders to assist with oxygenation in obtunded, critically ill patients. There are few recorded NPA complications.

**Case Report:**

We describe a unique case in which a patient went multiple days with recurrent symptoms of upper airway obstruction secondary to retained NPA.

**Discussion:**

Nasopharyngeal airways may be uniquely prone to being displaced and retained due to their use in emergent situations, their small size, and time of insertion in the field prior to emergency department (ED) contact where handoff is not often standardized.

**Conclusion:**

The use of large-flanged NPAs might reduce incidences of displacement into the nasal cavity. This case highlights the need for improved handoff communication between emergency medical services and ED staff, especially to account for all inserted devices to prevent foreign body retention.

## INTRODUCTION

Emergency providers and first responders often use airway adjuncts to assist with oxygenation in obtunded, critically ill patients. These adjuncts also assist ventilation and improve possible airway obstruction. The nasopharyngeal airway (NPA), or “nasal trumpet,” is one such device. Made of soft plastic or rubber, it is inserted into the naris to assist in maintaining oxygenation in the upper airway. It can be kept in place while endotracheal intubation is being performed to increase passive apneic oxygenation. The NPA is soft with a beveled tip to reduce trauma during insertion. The flared end on the opposite end is designed to prevent displacement of the NPA deeper into the nasopharynx where there would be difficulty in retrieving it.^1^ The size, design, and malleability of NPAs greatly vary, depending on the manufacturer. In particular, there is great difference in the flared end, with some having a large flange while others small. When inserting, the beveled tip of the NPA should lie no lower than the uvula.^1^

There are few recorded NPA complications. Perhaps the most clinically observed complication is stimulation of the gag reflex, along with epistaxis secondary to mucosal injury. A literature search revealed two reports of intracranial NPA placement in patients with basilar skull fractures, much like cases detailing nasogastric tube malposition.^2^ Additionally, we found a report of a middle turbinate fracture secondary to NPA placement resulting in severe epistaxis.^3^ In the case report presented here, we describe the retention of an NPA as a nasal foreign body (FB) causing acute upper airway obstruction. After an extensive search on PubMed, we found only one similar case report on this topic; however, this issue has not been discussed in the emergency medicine literature. In that earlier case, a retained NPA in the anterior nasopharynx for nearly 20 months was found to be causing dysphagia and odynophagia.^4^ Importantly, our case is unique in that the patient went multiple days with recurrent upper airway symptoms, including dyspnea, choking, and desaturation events on the monitor.

Considering that emergency providers and emergency medical services (EMS) frequently use these devices, we review this potentially serious complication and raise awareness for safer applications of NPAs.

## CASE REPORT

A 79-year-old male with a past medical history of type 2 diabetes, chronic bronchitis, coronary artery disease, and hypertension, presented with county EMS in acute respiratory failure. Per report at the scene, the patient was found unconscious and received 1 milligram of Narcan and atropine for bradycardia. On arrival the patient was receiving assisted ventilation via bag-valve-mask (BVM). He was subsequently intubated. Intubation was without difficulty and was performed by direct laryngoscopy on the first attempt by the emergency physician. The patient was admitted to the medical ICU.

**Image 1:**
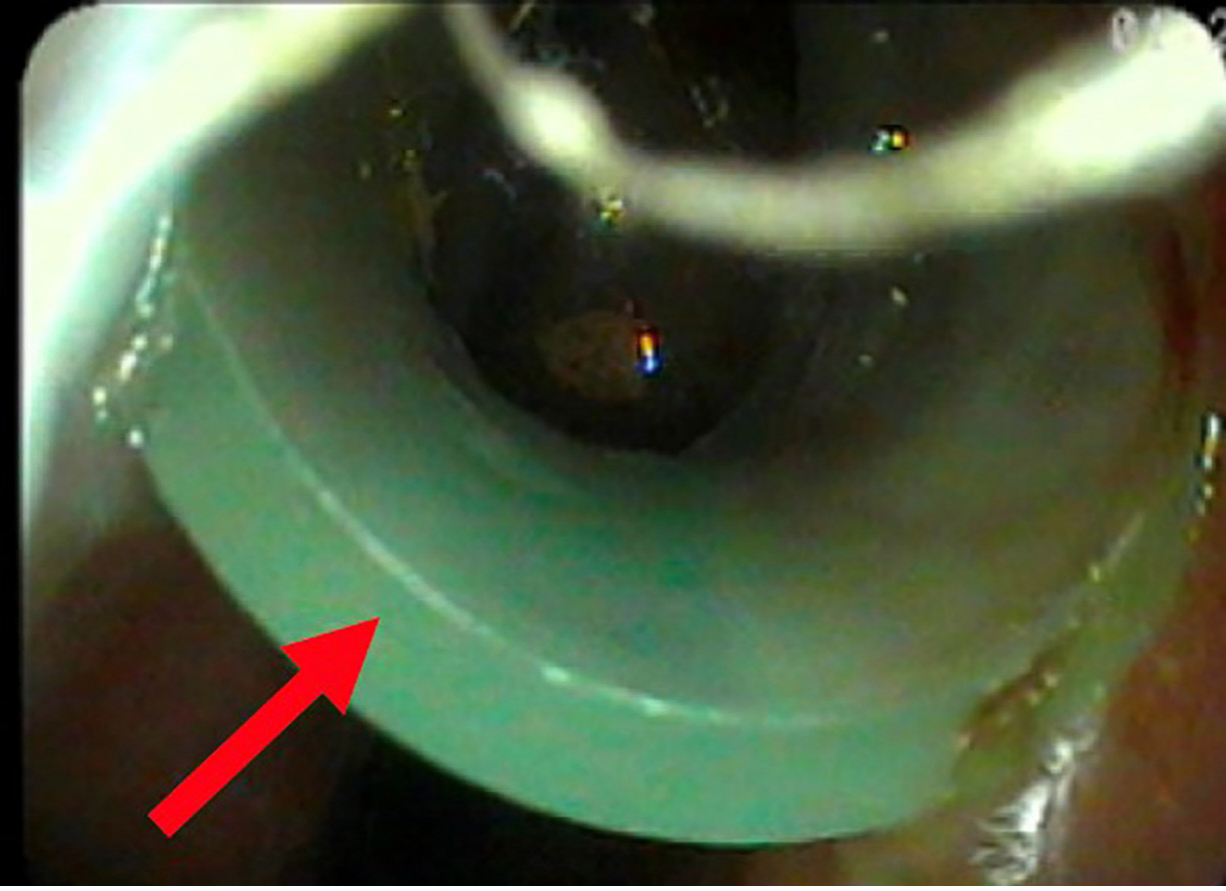
Nasal fiberoptic view of a retained nasopharyngeal airway (arrow) just distal to the right naris.

**Image 2:**
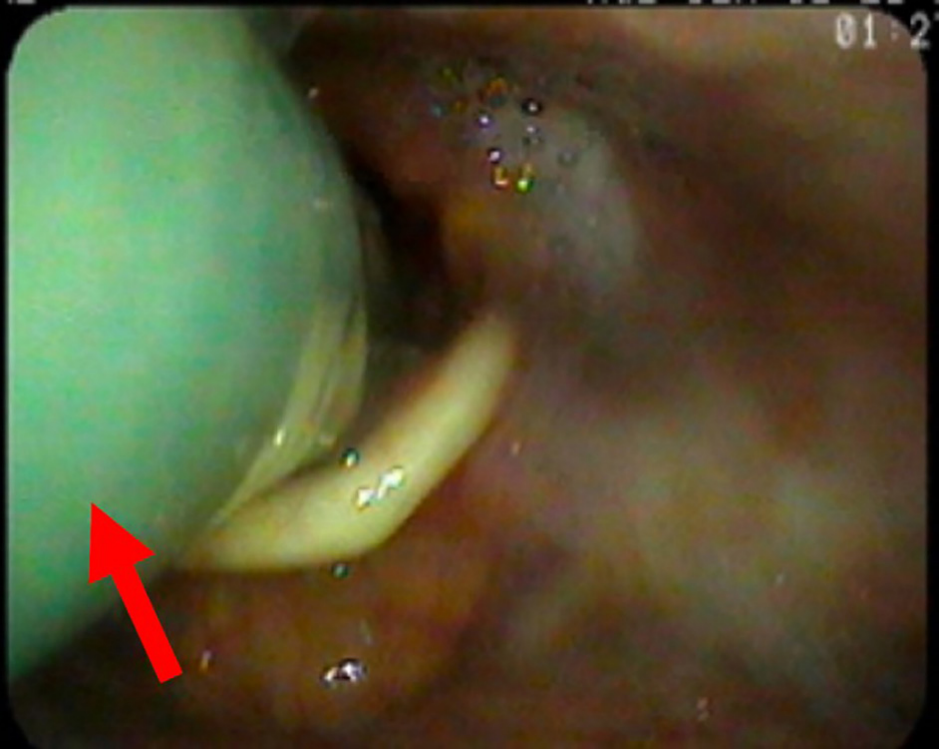
Nasal fiberoptic view of a retained nasopharyngeal airway (arrow) just superior to the larynx and posterior to the epiglottis.

Three days later the patient was extubated after improvement in his clinical condition. Following extubation, he continued to experience brief episodes of oxygen desaturations to the low 80s and endorsed the sensation of “something stuck in my throat.” He had a persistent cough, dyspnea at rest, and a feeling that he could not swallow. After speech therapy was consulted for evaluation, a FB was discovered during their nasal fiberoptic evaluation (Images 1–2). The FB was immediately superior to the larynx, and strongly suspected to be a cause of the patient’s symptoms. Otolaryngology (ENT) was consulted, and the FB was removed at bedside using Magill forceps without complication. After close examination, the FB was found to be an NPA. When reviewing medical records, it was found that the emergency physician and nurses had made no note of the NPA. It was concluded the NPA was likely placed by EMS in the field and had subsequently been displaced deep into the nasal cavity in transport and when ventilating via BVM. The patient continued the remainder of his hospital course without issue and was discharged one week later. He was not discharged with any new medications, and follow-up with ENT was arranged; however, there is no medical chart evidence that the patient followed up with ENT.

CPC-EM Capsule

What do we already know about this clinical entity?

*Nasopharyngeal airways (NPA), when retained as a foreign body, can cause lasting clinical detriment including recurrent discomfort and dyspnea*.

What makes this presentation of disease reportable?

*While considered safe they can be easily forgotten. A retained NPA caused lasting discomfort and likely increased the patient’s length of stay*.

What is the major learning point?

*An NPA, like any inserted foreign body, should be clearly listed during hand off*.

How might this improve emergency medicine practice?

*Clearer communication between first responders and emergency providers regarding all airway adjuncts could reduce complications with these tools*.

## DISCUSSION

This case highlights several safety issues that NPAs present to care teams, particularly EMS and emergency providers. The *Journal of the American Medical Association of Otolaryngology* published a report of a similar occurrence in 2019, which discussed a retained NPA that was placed by EMS in the field and ultimately remained in the patient for 20 months before surgical extraction. During the 20-month interim, the patient had dysphagia and odynophagia but continued to eat. He did not report symptoms of acute upper airway obstruction, such as dyspnea, cough, or stridor.^4^ In contrast, the patient in our case went multiple days with recurrent upper airway symptoms, including dyspnea, choking, and oxygen desaturation events on the monitor to the low 80s. Additionally, he could not tolerate swallowing well and did not pass his bedside- nursing swallowing evaluation; hence speech therapy was consulted. The NPA was found incidentally during the swallowing trial.

The flange size on a NPA may predispose it to displacement. In our case, the NPA that was removed had a small flange. This finding, along with the added difficulty of managing a critically ill patient in the field and mask seal during BVM ventilation, contributed to the NPA being dislodged and going unnoticed during intubation. One method to reduce NPA complications is immediate removal once a definitive airway is secured. Additionally, the NPA might have been the incorrect size for the patient. In general, the longer the NPA, the larger the diameter of the tube. For example, 8.0–9.0 centimeters (cm) is reserved for large adults, while 7.0–8.0 cm is standard adult size, and 6.0–7.0 cm is for smaller adults.^1^ Correct size can be estimated by placing the NPA next to the patient’s face with the flared end in line with the naris and the beveled tip toward the ear lobe. The goal is for the NPA to just reach the ear lobe.

This case also highlights the importance of closed-loop communication between healthcare teams. In the operating room, a rigid set of safety protocols exist to prevent iatrogenic-retained FBs. Our PubMed literature search revealed no such standardized processes in EDs, particularly during handoff from EMS to ED staff. Clear communication between the ED, EMS, and intensive care unit personnel about the types of airway adjuncts and assist devices in place are critical in reducing the likelihood of a retained FB. Iatrogenic FBs are a significant source of morbidity and cost to the patient. A study in the *Journal of the American College of Surgeons* found that retained FBs from a variety of situations are associated with higher cost of service and are more likely to occur in geriatric and obese patients.^5^

Nasopharyngeal airways are widely considered safe airway management tools that can be quickly deployed. These airway tools are portable and cost effective, and are actively used prior to establishing a definitive airway. Despite their widespread use, NPAs are not without complications, and there is always a potential risk for displacement and retention as a FB. To prevent this, clear communication that begins in the field with EMS, followed by proper handoff in the ED is essential. We also advise early removal of an NPA post-intubation, as well as the use of large-flanged NPAs to reduce chances of displacement into the nasal cavity.

## CONCLUSION

Nasopharyngeal airways are frequently used as a first-line airway adjunct in critically ill patients. They offer the unique benefit of easy placement and rapid portability from their size. However, NPAs may be uniquely prone to being displaced and retained due to their use in emergent situations, their small size, and time of insertion in the field prior to ED contact where handoff is not often standardized. Special consideration should be paid during patient handoffs as NPAs can prove quite difficult to identify at later stages in patient care. We advise that a more detailed documentation occur in the ED when a patient arrives with multiple devices in place, whether it be nasogastric or orogastric tubes, NPAs, oropharyngeal airways, intravenous access, or drains. The use of large-flanged NPAs might reduce incidences of displacement into the nasal cavity.
